# Intrapetrous internal carotid artery dissection and essential thrombocythemia: what relationship? A case report

**DOI:** 10.1186/1757-1626-1-354

**Published:** 2008-11-27

**Authors:** Daniele D'Ambrosio, David Della-Morte, Gaetano Gargiulo, Marianna Rossetti, Tatjana Rundek, Franco Rengo, Pasquale Abete

**Affiliations:** 1 Department of Internal Medicine, Cardiovascular Sciences, and Immunology, University Federico II, Naples, Italy; 2 Department of Neurology, University of Miami, Miller School of Medicine, Miami, FL – 33101, USA

## Abstract

Internal carotid artery (ICA) dissection is responsible for 10–20% of strokes in young and middle-aged patients. Isolated ICA dissection involving the intrapetrous carotid canal is particularly rare, and no case has been reported to describe an association between intrapetrous ICA dissection and essential thrombocythemia. We report a case of ischemic stroke in the presence of intrapetrous right ICA dissection and essential thrombocythemia. The diagnosis of essential thrombocythemia was performed by bone marrow biopsy. The essential thrombocythemia may cause endothelial dysfunction and predispose to vascular damage such as carotid artery dissection.

## Background

Internal carotid artery (ICA) dissection is responsible for less than 2% of all ischemic strokes, and for 10–20% of strokes in young and middle-aged patients, with a peak incidence in the fifth decade [1]. Isolated ICA dissection involving the intrapetrous carotid canal is particurarly rare [2]. Although not completely known, the main risk factors related to carotid artery dissection are genetic and environmental factors, traumatic events, cervical manipulations, migraine, recent infections, hyperhomocysteinemia, and hereditary connective-tissue disorders [3]. We report a case of ischemic stroke of a patient with intrapetrous ICA dissection and essential thrombocythemia.

## Case report

**Figure 1:**
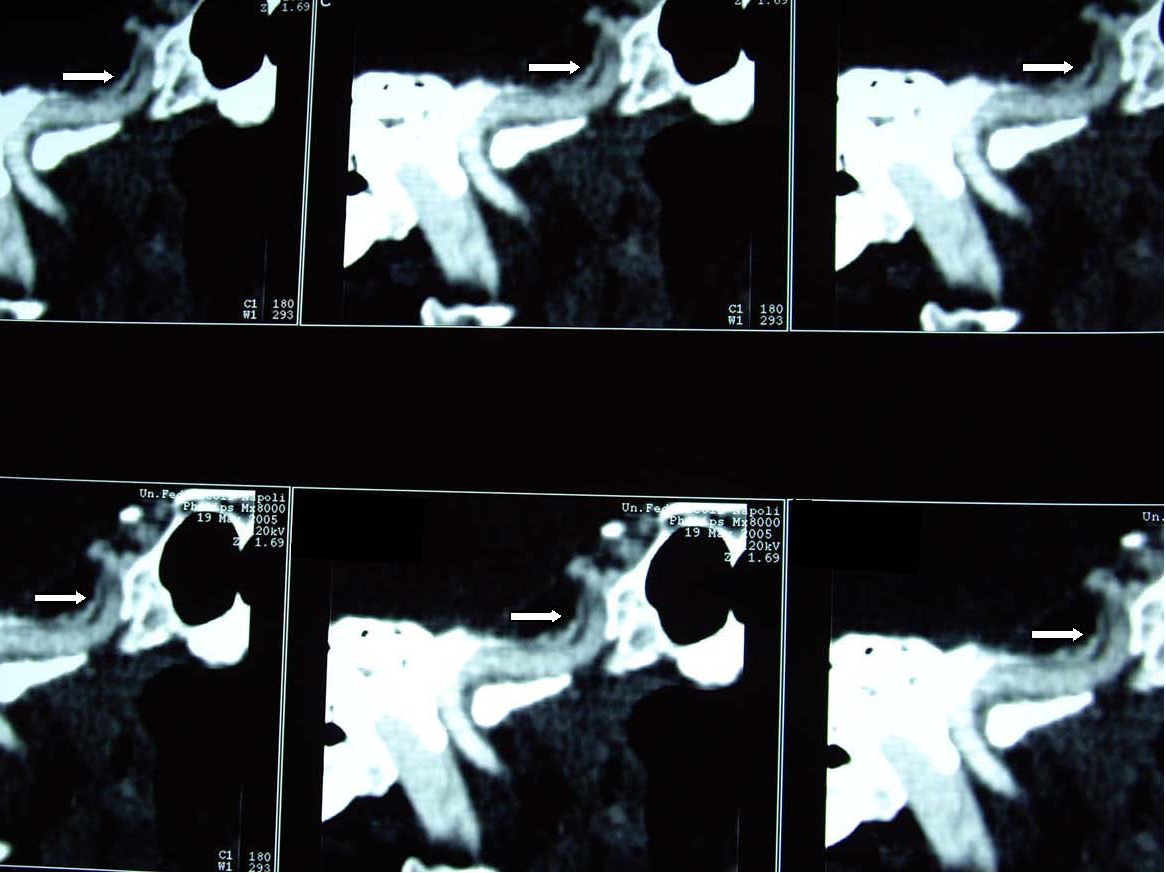
**ICA computed tomography angiography**. Internal carotid artery computed tomography angiography shows an irregular narrowing and an eccentric filling defect along the petrous portion of the right ICA suggestive of a dissection, as indicated by the white arrows in the sequence.

**Figure 2:**
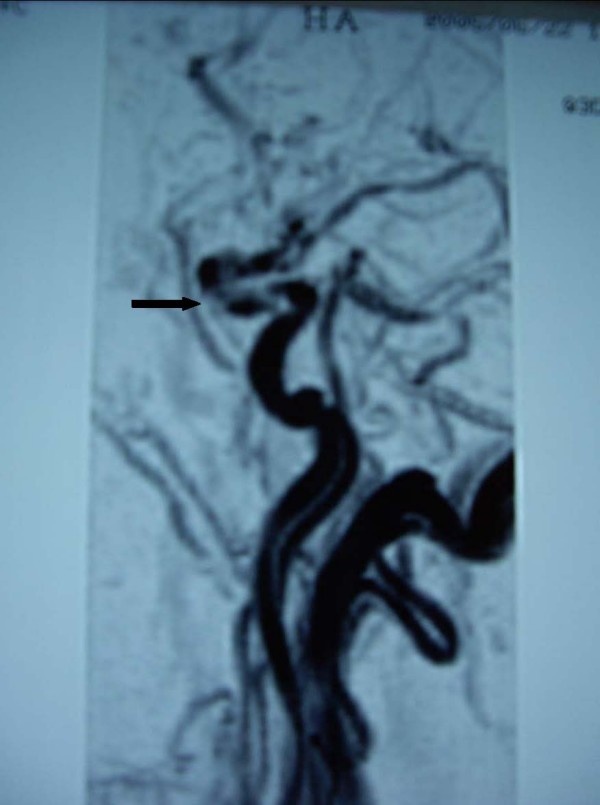
**Cervical magnetic resonance angiogram**. Cervical magnetic resonance angiogram at 1 month shows persistent filling defect of the right ICA, as indicated by the black arrow.

A 57-year-old woman was admitted to II° Policlinico hospital of Naples after an episode of left arm weakness, dysarthria and mouth deviation at left, occurred one week before admission. The patient had a medical history of paroxysmal supraventricular tachyarrhythmias and smoking. She denied any history of trauma and other cardiovascular risk factors. The patient was normotensive and on physical examination she presented a normal clinical state except for neurological evaluation that showed the persistence of left arm weakness and low facial paralysis with NIH stroke scale (National Institute of Health Stroke Scale, NIHSS) score of 3. Diagnostic evaluation included extensive laboratory blood tests (complete haematologic screening, routine biochemical profile, urinalysis), that were normal except for LDH (lactate dehydrogenase) increase and platelet count (426000/μL; range: 150000 – 400000/μL), chest roentgenography (normal), 12-lead electrocardiography (normal), transthoracic echocardiography (normal), carotid ultrasound, that revealed a fibrocalcific plaque at the left carotid bulb, without significant haemodynamic changes, brain magnetic resonance, that showed a cerebral ischemic at the posterior-frontal lobe of the right cortical hemisphere, and brain computed tomography (CT)-angiography, disclosing an irregular narrowing and an eccentric filling defect along the petrous portion of the right ICA suggestive of a dissection (Figure 1). Therefore she started a treatment with low molecular weight heparin (enoxaparin 4000 UI daily). A following month brain MR-angiography confirmed the dissection (Figure 2). She continued the anticoagulant therapy, waiting for a possible recanalitation with stent placement. Over the next three months, the patient experienced additional chest pain and one episode of parossistic atrial fibrillation. Electrocardiogram revealed signs of subendocardic ischaemia in the anterolateral leads. She was admitted to hospital, where a coronary and carotid angiography documented a high grade of stenosis of the proximal tract of anterior interventricular artery and a partial recanalitation of the dissection. A percutaneous transluminal coronary angioplasty (PTCA) and stenting of anterior interventricular artery was performed. The platelet count and other laboratory and instrumental exams were normal and she was started on platelet antiaggregation therapy with 100 mg for day of aspirin and 75 mg for day of clopidogrel, discontinuing enoxaparin. At 3-month follow-up a progressive increase of platelet count was observed until values reaches 872000/μL. At this point a haematological consult excluded other causes of thrombocytosis (reactive inflammatory thrombocytosis, iron deficiency, polycythemia vera, chronic myeloid leukemia, myelofibrosis and myelodysplastic syndrome). The bone marrow biopsy releaved a trilinear cellularity with megakaryocytes hyperplasia and dysplasia and excluded previous or other subtypes of Philadelphia Chronic myeloproliferative disease (CMPD) or myelodysplastic syndromes. Thus, the diagnosis of essential thrombocythemia (ET) was established. As accessory report, a portal vein thrombosis was disclosed at the abdominal ecography. The patient started therapy with hidroxyurea (15 mg/kg daily) and, at 1-year follow-up, she has normal values of platelet count and good clinical conditions, in absence of neurological symptoms and signs.

## Discussion

Spontaneous dissections of the ICA have often a good prognosis, but ischemic cerebrovascular events occur frequently: these lesions remain a major cause of stroke in young and middle-aged patients [1]. The immobility of the petrous portion fixed in the carotid canal makes the intrapetrous internal carotid artery dissection a rare event. Although thromboembolism is supposed to be the main stroke mechanism in ICA dissection [4], haemodynamic change can also play an important role, especially in cases of intracranial dissection [2]. Ischemic stroke attributed to essential thrombocythemia was found in 0.4% of cases, as reported in the Lausanne Stroke Registry [5]. Haematological disorders were identified as the definitive cause of cerebrovascular disease in 0.1% – 0.8% of cases in general stroke registries [5,6]. In our patient, the diagnosis of essential thrombocythemia was established after occurring episodes of stroke, right ICA dissection, coronary heart disease and portal vein thrombosis, all described as complications of this disease, except for dissection [7].

Essential thrombocythemia is a characterized by a high platelet count, originating from a pluripotent stem cell and it is diagnosed at a rate of about 2–3/100.000 individuals annually, with a slight female preponderance (1.5–2.1). The disease usually affects middle aged to elderly individuals, with an average age at diagnosis of 50–60 years. Approximately half of the patients are asymptomatic while the other half have vasomotor, thrombotic or hemorrhagic disturbances. Typical essential thrombocythemia is a Philadelphia bcr-abl negative (Ph1-) CMPD with a good prognosis and overall survival. For a long time, the Polycythemia Vera Study Group (PVSG) criteria for the diagnosis of ET have not included histopathological data. Recently, a new World Health Organization (WHO) classification for CMPD has been developed, incorporating clinical, laboratory and morphologic data along with fresh knowledge and techniques [8]. The clinical picture of essential thrombocythemia is dominated by a predisposition to vascular occlusive events and haemorrhages [7].

In our patient we observed a progressive increase of platelet count and various complications, typical of essential thrombocythemia. We performed a bone marrow biopsy and the study of *BCR-ABL *gene rearrangement and we excluded causes of reactive thrombocytosis. It is unclear whether there is a relationship between intrapetrous internal carotid artery dissection and essential thrombocythemia. An endothelial damage, due to the activation of leukocytes and the consequent release of elastase and alkaline phosphatase, is observed in essential thrombocythemia and it seems to play a major role about the pathogenesis of the thrombophilic state, including elevated levels of platelet-specific proteins, increased tromboxane generation and expression of activation-dependent epitopes on platelet surface [9]. The association between vasculitis disorders determining stroke, such as moyamoya disease, that affects the cerebral blood vessels mimicking sometimes a spontaneous internal carotid artery dissection on Doppler ultrasound, and essential thrombocythemia is described [10].

It is much more likely that thrombocytosis and elevated homocysteine, because they both increase thrombosis, make dissections more likely to be symptomatic, than that they actually predispose to dissection. Probably there are many patients with dissection that is asymptomatic; those with increased thrombosis are more likely to have symptoms and thereby be diagnosed.

## Conclusion

In summary, this case shows that essential thrombocythemia might predispose to vascular dysfunction and damage such as carotid artery dissection. Whether this association is casual or causal remains a matter of speculation. However, essential thrombocythemia should be excluded in the presence of carotid artery lesions and thrombocytosis.

## Consent

Written informed consent was obtained from the patient for publication of this Case report and any accompanying images. A copy of the written consent is available for review by the Editor-in-Chief of this journal.

## Abbreviations

CMPD: Philadelphia Chronic myeloproliferative disease; CT: computed tomography; ET: essential thrombocythemia; ICA: internal carotid artery; LDH: lactate dehydrogenase; MR: magnetic resonance; NIHSS: National Institute of Health Stroke Scale; Ph1-: Philadelphia bcr-abl negative; PTCA: percutaneous transluminal coronary angioplasty; PVSG: Polycythemia Vera Study Group; WHO: World Health Organization.

## Competing interests

The authors declare that they have no competing interests.

## Authors' contributions

DD and DDM conceived and designed the study; acquired the data; analyzed and interpreted the data; drafted the manuscript; made critical revision of the manuscript for important intellectual content. GG and MR helped to acquire the data, and made critical revision of the manuscript for important intellectual content. TR, FR, and PA drafted the manuscript; made critical revision of the manuscript for important intellectual content. All authors read and approved the final manuscript.
